# [Retracted] p68 prompts the epithelial‑mesenchymal transition in cervical cancer cells by transcriptionally activating the TGF‑β1 signaling pathway

**DOI:** 10.3892/ol.2024.14708

**Published:** 2024-09-30

**Authors:** Ming-Yi Li, Jin-Quan Liu, Dong-Ping Chen, Zhou-Yu Li, Bin Qi, Wen-Jing Yin, Lu He

Oncol Lett 15: 2111–2116, 2018; DOI: 10.3892/ol.2017.7552

Following the publication of this paper, it was drawn to the Editor's attention by a concerned reader that the scratch-wound assay data shown in Fig. 2C on p. 2114 were strikingly similar to data that had already appeared in a pair of previously published articles written by different authors at different research institutes. Owing to the fact that the contentious data in the above article had already been published prior to its submission to *Oncology Letters*, the Editor has decided that this paper should be retracted from the Journal. The authors were asked for an explanation to account for these concerns, but the Editorial Office did not receive a reply. The Editor apologizes to the readership for any inconvenience caused.

